# Secondary Metabolites from Natural Products: Extraction, Isolation and Biological Activities

**DOI:** 10.3390/molecules30214233

**Published:** 2025-10-30

**Authors:** Radosław Kowalski, Tomasz Baj

**Affiliations:** 1 Department of Analysis and Evaluation of Food Quality, University of Life Sciences in Lublin, 8 Skromna Str., 20-704 Lublin, Poland; 2 Department of Pharmacognosy with Medicinal Plants Garden, Medical University of Lublin, 1 Chodźki Str., 20-093 Lublin, Poland; tomasz.baj@umlub.pl

## 1. Introduction

Secondary metabolites constitute an extremely diverse class of compounds produced by plants, fungi, lichens, and microorganisms. Although they do not directly participate in basic life processes, they perform key ecological and adaptive functions: they facilitate chemical communication, defense against pathogens and herbivores, tolerance to environmental stress, and allelopathy. From the human perspective, many of them exhibit valuable biological activity (antibacterial, antifungal, antiviral, anti-inflammatory, antioxidant, and cytotoxic), which for decades has made natural products one of the most important sources of inspiration for pharmacotherapy and food technology. Synthetic reviews confirm that nature consistently provides new leading structures (leads) and drugs, ranging from antibiotics and anticancer agents to modern antibody–drug conjugates and therapies for lifestyle diseases [1,2,3].

From a scientific point of view, research on “Secondary Metabolites from Natural Products: Extraction, Isolation and Biological Activities” has several complementary goals. First, it allows for the scientific substantiation of traditional and ethnopharmacological uses of plants—from verifying historical indications to identifying the compounds responsible for the observed effect. Second, it enables the “rediscovery” of species described in historical sources and the exploration of biodiversity in new ecosystems (equatorial forests, Arctic and desert extremes, the deep sea, etc.). Third, it supports the search for alternative drugs, nutraceuticals, and functional ingredients through the integration of phytochemistry, microbiology, and molecular biology with modern analytics (LC–HRMS/MS, metabolomics, etc.). Finally, it offers pathways to sustainable applications in agriculture (biopesticides, allelochemicals), the food industry (natural preservatives, antioxidants, colorants, and emulsifiers), and even in industrial chemistry (biocatalysts, “green” reaction media, etc.). These lines of research are consistent with the European “Farm to Fork” strategy within the European Green Deal, which assumes, among other things, a 50% reduction in the use of and subsequent risk posed by chemical pesticides by 2030 [4,5,6,7,8,9].

In this Special Issue, we present cross-sectional examples of the implementation of these assumptions: from the optimization of extraction and comparison of methods for polyphenol-rich plant materials (e.g., *Scutellaria baicalensis*), through the use of supercritical techniques to obtain fractions with biological activity (e.g., *Sorbus aucuparia*), to the isolation and characterization of new structures from plants and fungi (meroterpenoids from *Garcinia caudiculata*, secobutanolides from *Lindera obtusiloba*, and metabolites of *Dentipellis fragilis* and endophytic *Xylaria*). Complementing these are review works (e.g., *Calendula officinalis* and *Foeniculum vulgare*, penicillides of *Penicillium/Talaromyces*, etc.), which organize knowledge and indicate research gaps, as well as studies in the area of ecophysiology and safety (allelopathy of lichens; Maillard reaction products as antioxidants). Together, they outline a map of current opportunities, from methodology and dereplication to applications in human health and agroecology.

### 1.1. Why Do We Study Secondary Metabolites?

Verification and consolidation of ethnopharmacology. Traditional knowledge is often a starting point for identifying bioactive molecules and mechanisms of action (e.g., alkaloids, terpenoids, and polyphenols). These studies transform empirical observations into evidence based on the standards of pharmacology and toxicology, serving the rationalization of the use of plant raw materials and the design of safe application forms [1,2,3].

Biodiversity as a resource for innovation. Extreme ecosystems (marine, Arctic, desert, etc.) and symbioses (endophytes, lichens, etc.) are “incubators” of unique structural scaffolds and biosyntheses. Maintaining, studying, and responsibly utilizing this biodiversity increases the chances of breakthrough discoveries [1,2,3].

Therapeutic and antimicrobial alternatives. In an era of antibiotic resistance and chronic noncommunicable diseases, natural products provide new points of engagement (targets) and pharmacophores, often with a multidirectional activity profile, which is advantageous in complex diseases [3].

Biocontrol and agroecology. Natural phytotoxins and biopesticides (including those of microbiological origin) can fit into integrated pest management (IPM), reducing environmental burden and the risk of pesticide residues in food. The development of this area supports the implementation of the Green Deal’s goals [4,5,6,7,8,9].

Functional ingredients and “clean labels.” The growing consumer demand for natural antioxidants, colorants, and preservatives directs attention to polyphenols, carotenoids, and phenolic metabolites with confirmed antioxidant and health-promoting activity, which are capable of replacing synthetic additives [10].

Sustainable media and catalysis. Research seeks “green” solvents and user-friendly technologies (e.g., pressurized water, supercritical CO_2_, and natural eutectics) that reduce emissions, energy consumption, and waste, without compromising extract quality [11,12,13,14].

### 1.2. From the Biological Sample to the Molecule: Modern Approaches to Extraction and Isolation

A key stage is the extraction and fractionation of complex biological matrices. Conventional techniques (maceration, percolation, Soxhlet) remain useful but can be material- and solvent-intensive. Therefore, “green extraction” techniques are increasingly used, including extraction with supercritical CO_2_ (SFE), ultrasound-assisted extraction (UAE) and microwave-assisted extraction (MAE), pressurized liquid/accelerated solvent extraction (PLE/ASE), and pressurized hot water extraction (PHWE). These techniques all display improved efficiency and selectivity with a smaller environmental footprint and better compatibility with downstream analysis [11,12,13,14]. For example, SFE–CO_2_ is nontoxic and nonflammable and allows the “tuning” of solvent strength with pressure/temperature or a small addition of a polar modifier, which favors the isolation of both volatile and medium-polarity fractions. PHWE uses water above 100 °C under pressure that keeps it in a liquid state. Under such conditions, the dielectric constant and viscosity decrease, while diffusivity increases, meaning that water “behaves” more like an organic solvent, effectively extracting numerous metabolites (including phenolic ones) without the use of classical solvents [12,13].

Natural deep eutectic solvents (NADESs) are another promising solvent platform, characterized as mixtures of inexpensive, biodegradable components (e.g., choline, organic acids, sugars, etc.) with a wide solubility window, capable of selectively extracting polyphenols or alkaloids, often without the need for subsequent desolventization [11,15,16]. In this context, glycerol is sometimes considered an environmentally friendly component of extraction systems—mentioned in this study only as an example among “green” media.

The choice of extraction method and medium should be based on the research objective (chemical profile vs. bioactive fraction), the nature of the matrix (content of lipids, hydrolytic enzymes), the thermal sensitivity of the compounds, and downstream requirements (e.g., compatibility with LC–MS/MS). In practical terms, the principles of “green extraction” call for minimizing solvent toxicity, energy consumption, and waste while maintaining the quality and reproducibility of analytical data.

### 1.3. From Extract to Annotated Chemical Space: Analytics and Dereplication

Advances in analytical methods enable the rapid “mapping” of chemical composition and the prioritization of fractions. LC–HRMS/MS combined with bioinformatics has revolutionized metabolite annotation, driving a shift from classical dereplication (comparing spectra with databases, structure prediction) to molecular networking (GNPS) and its feature-based version (FBMN), which connect spectra into similarity networks, facilitating the identification of isomers and biosynthetic families and the integration with quantitative data. These tools shorten the path from the “dark chemical matter” to structural and biological hypotheses and also limit the repeated “discovery” of known compounds [17,18,19,20].

### 1.4. Biological Activity and Pathways to Applications

The evaluation of bioactivity includes standardized antibacterial/antifungal tests, antioxidant assays (e.g., radical systems, chemiluminescence, etc.), anti-inflammatory assays (mediators, cytokines, NO, etc.), cytotoxicity tests, and functional tests (target enzymes, receptors, etc.). In agriculture, biopesticides and allelochemicals are attracting increasing attention, which—with appropriate standardization and risk assessment—can support integrated plant protection programs and the implementation of public policy goals to reduce chemical pesticides [4,5,6,7,8,9]. In food technology, the priority is to replace synthetic additives with natural antioxidants and preservatives, which is facilitated by a growing body of data on the effectiveness and stability of polyphenols in various matrices [10].

## 2. Plant Secondary Metabolites

### 2.1. Polyphenols and Flavonoids

Polyphenols are widely distributed in the plant world. Tenório et al. [Contribution 1] isolated, among other substances, myricitrin, gallic acid, and ellagic acid from the leaves of *Eugenia uniflora* L. (Myrtaceae), which showed significant antifungal activity against *Candida albicans*, *C. glabrata*, and *C. auris* strains. The strongest effect was exhibited by fractions rich in myricitrin and ellagic acid (MIC 62.5–500 µg/mL). The isolated single constituents were characterized by weaker activity than the fractions of constituents, which indicates an important role of interactions among constituents in the complex mixture. *Eugenia uniflora* L. is a source of natural constituents characterized by anti-*Candida* potential, which is important in the context of the growing resistance to classical antifungal drugs.

Dzięcioł et al. [Contribution 2], in a study on the roots of *Scutellaria baicalensis* Georgi. (Lamiaceae), applied five extraction techniques, which made it possible to obtain ethanolic extracts differing in terms of the content of phenolic compounds and flavonoids. The highest antioxidant activity was exhibited by extracts after reflux extraction and Soxhlet extraction, and this activity was correlated with the highest content of the studied groups of biologically active compounds, while the extraction yield depended on the conditions used. The most important compounds identified in the extracts were flavonoids characteristic of this species, wogonin and oroxylin A, as well as other hydroxyflavone derivatives. Dzięcioł et al. demonstrated that in extraction using a Soxhlet apparatus, 5-hydroxymethylfurfural was formed, which constitutes a potentially undesirable constituent, and therefore they recommend reflux extraction with ethanol for 2 h as the most effective and safe method to obtain extracts.

Ryszczyńska et al. [Contribution 3] assessed the potential of rowanberry (*Sorbus aucuparia* L., Rosaceae) fruits as a natural agent for the biological protection of plants against pathogens of the genus *Fusarium* (*Fusarium proliferatum* and *F. culmorum*), including the effect on mycotoxin biosynthesis by these phytopathogens. Extracts were prepared by means of supercritical fluid extraction (SFE, using CO_2_ with methanol as a co-solvent) under a variety of different temperature and pressure conditions. The rowanberry extracts showed diverse activity; for example, extracts obtained under conditions of 70 °C/300 bar exhibited the strongest inhibitory effect on the growth of *F. proliferatum* and on the reduction in ergosterol content, whereas in the case of *F. culmorum*, no clear reduction in colony growth was recorded, but the decrease in ergosterol content indicated a limitation of mycelial biomass. The *Sorbus* extracts exhibited different inhibitory–stimulating effects on the biosynthesis of various mycotoxins depending on the *Fusarium* species, indicating the potential of this plant in the biological protection of crops. The tested extracts from *Sorbus aucuparia* may be a promising tool for the biological control of *Fusarium* in cereal crops.

### 2.2. Terpenoids and Meroterpenoids

Valmiki et al. [Contribution 4] isolated two meroterpenoids from the leaves of *Garcinia caudiculata* Ridl. (Clusiaceae) from the island of Borneo, including, for the first time, caudiquinol containing a full geranylgeranyl chain, and a previously described benzofuranone lactone. The structures of the two isolated constituents were established (NMR and MS). The crude dichloromethane extract showed moderate antibacterial activity against a *Staphylococcus aureus* MSSA strain; however, the pure constituents did not exhibit significant antibacterial activity, and no cytotoxic effect was observed against a lung cancer cell line.

Yang et al. [Contribution 5], from the stems of *Lindera obtusiloba* Blume (Lauraceae), used traditionally in Oriental medicine as an anti-inflammatory and hepatoprotective agent, isolated new secobutanolides and determined their structures based on the analysis of spectroscopic data. The biological activity of the isolated secobutanolides was evaluated against bone-marrow-derived dendritic cells stimulated with lipopolysaccharide (LPS). The effect on the production of pro-inflammatory cytokines IL-6 and IL-12 p40 with anti-inflammatory action was assessed. Three of the compounds studied showed a strong inhibitory effect on cytokine synthesis and at the same time did not show cytotoxicity at the tested concentrations. The isolated constituents constitute a starting point for the development of new anti-inflammatory compounds.

Vella et al. [Contribution 6] prepared a review devoted to *Calendula officinalis* L. (Asteraceae) and *Foeniculum vulgare* Mill. (Apiaceae), which, from a synthetic perspective, indicates a rich set of biologically active constituents in these species: phenols (including phenolic acids and flavonoids), terpenes (monoterpenes, sesquiterpenes, triterpenes, and carotenoids), and alkaloids. Both species, present in phytotherapy, cosmetics, and dietetics for centuries, are currently included in many pharmacopeias. *C. officinalis* is characterized by antioxidant, anti-inflammatory, antimicrobial, cytotoxic, hypoglycemic, nootropic, and anticancer properties. Of particular importance in this species are flavonoids (quercetin, rutin, luteolin, etc.) and triterpenoids (including faradiol and calendula saponins)—these compounds are responsible for anti-inflammatory effects, wound healing, and liver and kidney protection, as well as potential antiviral properties (HSV, HIV). In turn, *F. vulgare* is valued for its aromatic fruits and essential oil. It shows antioxidant, antibacterial, antifungal, antiviral (HSV-1, PI-3), anti-inflammatory, anticancer, hepatoprotective, cardioprotective, gastroprotective, antidiabetic, and estrogen-like activity. Traditionally, it has been used in the treatment of digestive disorders, respiratory diseases, and ocular ailments and as a spice. Both *C. officinalis* and *F. vulgare* are plants with great potential for further research and fuller use in health prevention and in the development of nutraceuticals, dietary supplements, and herbal medicines.

## 3. Fungal and Endophytic Metabolites

### 3.1. Wood-Inhabiting and Endophytic Fungi

Sum et al. [Contribution 7] studied metabolites occurring in cultures of the rare wood-inhabiting fungus *Dentipellis fragilis* (Pers.) Donk (Hericiaceae), among which the structures of five compounds were described, including two new substances, dentifragilone A and dentifragilone B, along with three known derivatives of benzoic acid and drimane. In microbiological tests, only the methyl ester of 4-chloro-3,5-dimethoxybenzoic acid showed moderate activity against *Staphylococcus aureus*, whereas studies on specific cell lines (mouse L929 fibroblasts and the KB3.1 cervical cancer line) did not observe cytotoxicity for these constituents. The results indicate that even rare temperate-zone Basidiomycota fungi can provide new, hitherto undescribed metabolites, although their biological activity is sometimes limited and requires further verification.

Zhang et al. [Contribution 8] conducted chemical studies on the endophytic fungus *Xylaria* sp. Z184 isolated from the leaves of *Fallopia convolvulus* (L.) Á. Löve of the Polygonaceae family. From a methanolic extract of the culture of this strain, three new pyranone derivatives—fallopiaxylaresters A, B, and C—and a new bisabolane-type sesquiterpenoid named fallopiaxylarol A were isolated and characterized. In addition, known compounds were identified in the extracts studied, including other pyronones, sesquiterpenoids, isocoumarin derivatives, and an allylaryl ether. Studies of biological activity showed that the crude extract of the fungus strongly inhibited nitric oxide (NO) production in RAW264 macrophage cells. Selected compounds (e.g., pestalotiopyrone M, xylariaopyrone A and H) exhibited only weak antibacterial activity against *Staphylococcus aureus*. *Xylaria* sp. Z184 constitutes a rich source of compounds with diverse chemical structures. The results obtained confirm the potential of endophytic fungi in the search for new metabolites with anti-inflammatory and antimicrobial properties.

### 3.2. Penicillium and Talaromyces

The review by Salvatore et al. [Contribution 9] discusses penicillides, a unique group of secondary metabolites of these fungi, whose prototypical representative is penicillide, first described in the 1970s in species of *Penicillium*. These compounds occur mainly in fungi of the genus *Talaromyces*, and sporadically in other *Ascomycota* and even in plant tissues. Penicillides are characterized by a depsidone structure with an eight-membered heterocyclic ring and various side substituents. In terms of their biological activity, penicillides exhibit a broad spectrum of effects, including antibacterial, antifungal, antimalarial, and antiplasmodial activity, as well as having cytotoxic effects against various cancer cell lines. They also exhibit other biochemical actions such as calpain inhibition (with potential relevance in the treatment of muscular dystrophies and neurodegenerative diseases), elastase inhibition (COPD therapy), the modulation of oxytocin binding, while also exhibiting anti-inflammatory activity. The authors emphasize the potential of penicillides as starting structures for creating semi-synthetic derivatives with greater potency, especially in the context of increasing antibiotic resistance and the growing importance of respiratory diseases.

### 3.3. Marine and Extremophilic Microorganisms

The study by Wu et al. [Contribution 10] concerned the Arctic strain *Streptomyces* sp. MNP-1, isolated from a sample of marine origin. In order to increase the diversity of metabolites, the OSMAC (“One Strain, Many Compounds”) strategy was used, consisting of culturing the microorganism under different conditions and on different media. In this study, 20 secondary metabolites were isolated and identified, some of which were reported for the first time in microorganisms. Biological tests confirmed that several of these compounds possess significant antibacterial (including against *Staphylococcus aureus* and *Escherichia coli*) and antifungal activity (e.g., against *Candida albicans*), whereas two compounds (phenazine and staurosporine) additionally showed moderate anticancer effects against lung (A549), breast (MCF-7) and liver (HepG2) cancer cell lines. The *Streptomyces* sp. MNP-1 strain is a promising producer of bioactive secondary metabolites, which may be of interest in the development of new antibiotics and potential anticancer drugs.

## 4. Lichen Metabolites and Allelopathy

Lichens are interesting symbiotic systems in which secondary metabolites play allelopathic roles. Bačkor et al. [Contribution 11] examined the effect of secondary metabolites of Australian lichens *Ramalina celastri* (usnic acid) and *Stereocaulon ramulosum* (a mixture of atranorin and perlatolic acid) on the growth and metabolism of the lichen photobiont *Asterochloris erici* grown under aposymbiotic conditions. Their study showed that the extracts acted phytotoxically, reducing the growth of photobiont cultures. The mixture of atranorin and perlatolic acid showed a stronger effect than usnic acid alone. Extracts from *S. ramulosum* caused greater damage to the photosynthetic system, a decrease in pigment content, an increase in lipid peroxidation, and a decrease in the levels of endogenous antioxidants. The changes also concerned the metabolism of organic acids, which was considered a sensitive marker of phytotoxicity. This study confirms that lichen secondary metabolites act as allelochemicals, regulating the balance between the partners of the lichen symbiosis and they can significantly affect ecosystem functioning.

## 5. Maillard Reaction Products as a Source of Bioactive Compounds

Products of the Maillard reaction (MRPs), formed during the process of reducing sugars with amino acids, are known for their role in forming the color and flavor of food. Bolchini et al. [Contribution 12] developed and optimized a method for the determination of antioxidant Maillard reaction products (KAMs—“known antioxidant MRPs”) using liquid chromatography coupled with high-resolution mass spectrometry (HPLC-HRMS), applying different combinations of 20 amino acids and 6 reducing sugars depending on pH and reaction time. The authors showed that neutral and alkaline pH favored the formation of compounds with antioxidant activity, with optimal conditions obtained at pH 7. The highest production of antioxidant compounds was recorded in reactions involving threonine and disaccharides (maltose, lactose, etc.). The results of this study indicate the potential possibility of using the Maillard reaction to obtain natural antioxidants for use in the production of functional foods.

## 6. Hemp Extracts and Functional Applications

Industrial hemp varieties (*Cannabis sativa* L., Cannabaceae Endl.)—traditionally used for the production of fibers and seeds—can be a valuable source of bioactive compounds with potential applications in the prevention of neurodegenerative diseases and in the production of functional foods. Sip et al. [Contribution 13] studied extracts from *C. sativa* obtained by means of supercritical CO_2_ extraction (SFE) and then combined them with prebiotic carriers, namely dextran, inulin and trehalose, creating three delivery systems for active substances. HPLC analyses showed the presence of key cannabinoids (CBD) and trace amounts of tetrahydrocannabinol (THC). All extracts exhibited the ability to neutralize free radicals, whereas in neuroprotective tests, the extracts inhibited acetyl- and butyrylcholinesterase (AChE and BChE). The obtained systems of hemp extracts with prebiotic carriers supported the growth of beneficial gut bacteria (*Bifidobacterium*, *Lactobacillus*, *Faecalibacterium*), while maintaining antioxidant and neuroprotective properties. The combination of hemp extracts with prebiotic substances opens up new possibilities for developing innovative nutraceutical delivery systems.

## 7. Synthesis of Knowledge and Perspectives

The analysis of the entire set of studies allows several key trends to be distinguished:

- Diversity of sources—plants, fungi, lichens, endophytes, extremophilic microorganisms, etc.;
- New structures—meroterpenoids, secobutanolides, pyranones, penicillides, etc.;
- Methodological progress—supercritical CO_2_ extraction, optimization of conventional solvent extraction, HPLC-HRMS profiling, etc.;
- A wealth of biological activities—antibacterial, antifungal, antioxidant, anti-inflammatory, anticancer, etc.;
- Application possibilities—functional foods, nutraceuticals, crop biocontrol, new drugs, etc.

It is worth emphasizing the role of the review papers presented in this Special Issue, which organize knowledge about selected species and classes of compounds, supplemented by original research, presenting new experimental data.

## 8. Conclusions

Secondary metabolites remain an inexhaustible source of inspiration for the natural and applied sciences. Advances in extraction techniques, bioanalysis and structural characterization accelerate the discovery of compounds with potential pharmaceutical, agricultural, and industrial applications. This Special Issue of *Molecules* emphasizes the importance of integrating phytochemistry, microbiology, and biotechnology in order to fully utilize natural resources in innovations consistent with the principles of sustainable development. The studies collected in this Special Issue implement the objectives indicated in the Introduction: they verify ethnopharmacological premises (the richness of polyphenols and terpenoids), explore biodiversity (tropical plants, wood-inhabiting fungi and endophytes, marine microorganisms, etc.), develop ‘green’ extraction and dereplication methods (SFE, LC–HRMS/MS, network approaches, etc.), and also propose directions for practical implementations, from biocontrol and agroecology to functional foods and potential lead compounds in drug discovery.

## Data Availability

No new data were created or analyzed in this study. Data sharing is not applicable to this article.
